# Domestic Summary

**Published:** 1839-05-25

**Authors:** 


### DOMESTIC SUMMARY.

*A Fœtus with two heads.*—On the morning of the 25th of April last, I was called to visit Mrs. D., in labour with her fourth child, about four miles from this village. I arrived about 1 o'clock, A. M., and found her in severe labour. She informed me her pains had been increasing for about two hours. On examination, after placing her on her left side, I found the membranes distended with their fluid; but I could not discover the fœtus by the touch. I avoided rupturing the membranes as much as possible, trusting to the fœtus to settle in the fluid, and suffered them to give way. The head on the right side presented, with the face to the os pubis. With some difficulty the head passed, but the labour did not propel it further. On further examination, I found the funis passing over the shoulder on the left side. In search for the shoulder, I found a hard substance, resembling a head, and soon discovered a union of the two. I succeeded in raising it, and by the assistance of a pain lodged it on the side of the neck of the first head, and in eight or ten minutes delivered my patient of a stillborn male child, that would weigh about twelve pounds, with two regular heads and necks, of the usual size, and with fair features. No difficulty with the placenta. Mrs. D. was in labour about one hour and a quarter after I arrived at the house. She is convalescing as fast as women generally do after severe labour.

The child appears broader across the sternum than children usually are. The necks are at a suitable distance apart, sufficiently so to allow each head to stand erect without interfering with the other. At the junction of the shoulders, in place of one or two regular arms, there is a straight, firm bone, of a conical shape, about five or six inches in length, apparently in a socket, base down, regularly articulated to the transverse spinous process junction of the two spines. There are three clavicles; the two lateral arms are in their proper position. There are evidently two spines extending to the os sacrum, and connected by the transverse processes of the vertebræ of the back. The lower extremities are natural.

I have stated the case briefly, as it appears from external examination. It seems to be similar to a deformed fœtus reported by Professor W. E. Horner, of Pennsylvania, in the 8th volume of the American Journal of Medical Sciences, in 1831.

I have nothing further to add, unless I inform you that I invited one of my medical friends of the village to accompany me in the afternoon to examine the child. We succeeded in prevailing on the parents to permit us to take it and preserve it in a careful manner, for the inspection of the medical faculty. Wm. Baker.

*Oswego, N. Y.*, May 4th, 1839.

*Boston Med. and Surg. Journ.*

---

*Extract of Red Clover (Trifolium pratens.)*—At the Shaker village of Canterbury, N. H., this article has been prepared several years, and with the families of that community, and others, who have received decidedly beneficial results from its application, it has a high reputation. We are assured by Dr. Corbett that on ulcerated surfaces, deep, ragged-edged and otherwise badly conditioned burns, there is nothing to be compared with it. In connection with a peculiar soothing property which it imparts to an inflamed, irritable sore, it proves an efficacious detergent, and promotes a healthful granulation. As the process of making the extract is exceedingly simple, the material being abundant both in the field and by the way side, it is worth the immediate attention of general practitioners, from some of whom we hope to hear a good report of its medicinal virtues.

